# Colden’s Liebig’s Liquid Extract of Beef

**Published:** 1879-06-20

**Authors:** William Alex. Greene

**Affiliations:** Macon, Ga.


### *COLDEN'S LIEBIG'S LIQUID EXTRACT OF BEEF.*

BY WILLIAM ALEX. GREENE, M.D., OF MACON, GA.

It is impossible to estimate how much is due to improved and skilled pharmacy of the present day for the increased efficiency of our remedies, especially of that class of remedial agents known as nutritive tonics and stimulants. From their action on the digestive organs it would appear that the more nearly tonic medicines approximate to the aliment which would be the most easily digested, and the most decidedly nutritious, the greater the influence they would possess. They should never be of a nature to produce any inordinate excitement, for the reaction or exhaustion that would follow upon the stimulus would be more hurtful than any beneficial influence they could exert. It is a slow, steady and uniform operation that is required; and the greater the delicacy of the constitution for which we are called to prescribe, the more careful must we be in the quality, quantity and mode of operation of our food, tonics and stimulants.

Colden's Liebig's liquid extract of beef and tonic invigorator is recognized and prescribed by the leading physicians of the North and in the South as just the remedy to fill all these indications. It is composed of the purest extract of beef (liquid), without any fat, bone or sinew, and after Prof. Liebig's process, together with, and in addition to, iron, the alkaloids of chinchona, gentian, pure wine and aromatics. It is not a patent or proprietary medicine, as all physicians are furnished with formula if desired.

There are many beef extracts in the market, but none contain the important and essential properties of this preparation, which makes it, at the same time, a nutritive tonic, stimulant and alterative. Since the invention of the "Extract of Meat," by Liebig, there has been much discussion *pro* and *con*, concerning its physiological action and nutritious value. But the practical uses which have been made of the invention speaks in high favor of its great value and importance; neither have we waited in vain for the experimental demonstration that this extract is capable of replacing the valuable nutritious parts of meat. Liebig's extract consists essentially of two kinds of chemical substances, namely : Mineral salts, mostly of potash and extractive matters of meat, and that it is essentially promotive in the formation of the blood and tissues, and exercising also an exciting influence upon the activity of the heart,

It may not be uninteresting to be informed that this valuable liquid beef preparation was produced at the solicitation of the German authorities just before the late Franco-Prussian war, and that it proved of immense service during the severe campaigns of that war, being sufficiently strengthening to sustain the soldiers under circumstances when *solid* food could not be obtained, and in cases among the sick and wounded it proved an unspeakable blessing.

To bring results of this valuable liquid preparation nearer home, I will state that I have tested its virtues and efficiency in my private practice in cases of general *debility* and *depression* of the vital organs, when medicine had proven more than useless; also in cases of dyspepsia and the multitudinous nervous affections resulting from it, with complete loss of appetite and constipation of the bowels, and particularly when *delicate females*, were the unfortunate subjects of such troubles —often with infants to nourish. I have found it the best remedy I have ever used in chronic alcoholism, when the stomach is always irritable, and food required to nourish and invigorate the drooping strength and nervous depression, at the same time appeasing the thirst for more alcohol.

This preparation of T. Colden's must not be confounded with the ordinary liquid extracts of beef made by druggists generally, the fault of which is that they are made from meat which has undergone *chemical changes* and rank, as Dr. Newman has remarked, only as stimulants. But this preparation submitted to the medical profession is citrate of iron, alkaloids of chinchona flava, extract of gentian, with extract of beef (Baron Von Liebig's process), flavored with aromatics, and is a stronger extract than we ever get in drug stores according to ordinary formulæ. This is a reliable preparation, and supplies a want as an invigorator and nutritious food tonic long desired by the profession.—*Md. Med. Journal.*

